# Lanthanoids Goes Healing: Lanthanoidic Metallopolymers and Their Scratch Closure Behavior

**DOI:** 10.3390/polym12040838

**Published:** 2020-04-06

**Authors:** Stefan Götz, Stefan Zechel, Martin D. Hager, Ulrich S. Schubert

**Affiliations:** 1 Laboratory of Organic and Macromolecular Chemistry (IOMC), Friedrich Schiller University Jena, Humboldstr. 10, 07743 Jena, Germany; goetz.stefan@uni-jena.de (S.G.); stefan.zechel@uni-jena.de (S.Z.); 2 Jena Center of Soft Matter (JCSM), Friedrich Schiller University Jena, Philosophenweg 7, 07743 Jena, Germany

**Keywords:** metallopolymers, self-healing, lanthanoids, supramolecular polymers

## Abstract

Metallopolymers represent an interesting combination of inorganic metal complexes and polymers resulting in a variety of outstanding properties and applications. One field of interest are stimuli-responsive materials and, in particular, self-healing polymers. These systems could be achieved by the incorporation of terpyridine–lanthanoid complexes of Eu (III), Tb (III), and Dy (III) in the side chains of well-defined copolymers, which were prepared applying the reversible addition fragmentation chain-transfer (RAFT)-polymerization technique. The metal complexes crosslink the polymer chains in order to form reversible supramolecular networks. These dynamics enable the self-healing behavior. The information on composition, reversibility, and stability of the complexes was obtained by isothermal titration calorimetry (ITC). Moreover, self-healing experiments were performed by using 3D-microscopy and indentation.

## 1. Introduction

As a special class of polymers, metallopolymers are an important representative of materials in polymer science. The incorporation of metal complexes into polymeric structures results in beneficial combinations of both classes (inorganic complexes and polymers) and leads to unique properties of the resulting materials [1]. Consequently, a wide range of innovative applications were already achieved, ranging from catalysis [2], over medical applications like polymeric drug delivery systems [3], to organic light-emitting diodes (OLEDs) based on polymers [4]. By adjusting the structure of the metal–ligand interaction further properties can be generated. Thus, stimuli-responsive [5] and self-assembling polymers could be generated [6,7]. In particular, stimuli-responsiveness on several stimuli like heat or light enables shape-memory polymers [8,9,10] as well as materials featuring self-healing ability [11,12].

The current study focuses on metallopolymers featuring the ability of self-healing. This kind of materials was firstly described in 2011, in which light irradiation was utilized in addressing the metal–ligand interaction enabling scratch closure behavior [13]. However, due to the light, a heating of the material up to 220 °C was achieved that generates the required mobility for healing of damages. For this purpose, 2,6-*bis*(1’-methylbenzimidazolyl)pyridine ligands were *α,ω*-functionalized on poly(ethylene-*co*-butylene) copolymers. The subsequent complexation with either zinc (II) or europium (III) ions resulted in reversible, photo-responsive bonds. Next to light as external trigger, several studies focused on the temperature-responsive self-healing of metallopolymers. Therefore, the incorporation of terpyridine into polymers was a promising strategy. In literature, several examples of polymers with terpyridine complexes of metals like cadmium (II) [14], manganese (II) [15], or platinum (II) [16] have been reported. The healing behavior could be further enhanced through weak coordinating counter ions like acetate [14,15], chloride [14,15], or pyridine derivates [16]. These weak ligands improved the self-healing ability resulting in self-healing temperatures below 100 °C [15]. The necessity of the incorporation of the supramolecular binding moiety, *i.e.*, metal complexes, could also be proven by preparing an irreversible covalent polymer network, which only differs by the crosslinking unit—irreversible covalent vs. reversible metal–ligand interaction [15]. In this case, it could be shown that the covalent network is unable to heal mechanical damage and that the reversible binding motif is required for enabling healing. Furthermore, metallopolymers were studied regarding their thermal behavior using Raman spectroscopy [10]. In this case, no significant structural changes could be observed leading to the assumption that the mechanism is based on exchange reactions.

In the present study, we aim to expand the variety of self-healing metallopolymers based on terpyridine complexes by the coordination of the different lanthanoid ions, namely europium (III), terbium (III), as well as dysprosium (III). It is well known that complexes of these metals have luminescent properties [17,18,19,20]. Furthermore, Chen et al. have shown that terpyridine complexes of Eu (III) and Tb (III) also feature stimuli-responsiveness due to the dynamic metal ligand coordination [21]. Consequently, self-healing ability can also be expected, which will be investigated in the current study by utilizing isothermal titration calorimetry (ITC), microscopy, indentation and NMR spectroscopy. Consequently, the study will provide more information on the self-healing ability of metallopolymers based on terpyridine complexes and will presumably enable functional self-healing materials later on.

## 2. Experimental Section

### 2.1. Materials and Methods

All chemicals were purchased from Acros Organics (Geel, Belgium), Aldrich (Darmstadt, Germany), Alfa Aesar (Kandel, Germany), Fluka (Darmstadt, Germany), HetCat (Basel, Switzerland), Biosolve (Dieuze, France), Merck (Darmstadt, Germany) and TCI (Eschborn, Germany) and were used without further purification unless otherwise specified. Triethylamine was dried over calcium chloride and kept under nitrogen using standard Schlenk techniques. Butyl methacrylate (BMA) was passed over a short neutral aluminum oxide plug. Chromatographic separations were performed either with silica gel 50 from Merck or with neutral aluminum oxide from Molekula. The reaction process was monitored by thin layer chromatography (TLC) using aluminum sheets precoated with silica gel 60 F_254_ or aluminum oxide 60 F_254_.

^1^H and ^13^C NMR spectra were recorded on a Bruker AC 250 (250 MHz) (Bruker, Billerica, MA, USA), on a Bruker AC 300 (300 MHz) spectrometer (Bruker, Billerica, MA, USA), at 298 K and chemical shifts are reported in parts per million (ppm, *δ* scale) relative to the signal of the applied solvent. Coupling constants are given in Hz. Elemental analysis was performed on a Vario El III (Elementar, Langenselbold, Germany) and an Euro EA EKATech elemental analyzer (HEKAtech GmbH, Wegberg, Germany) as well as halogen analysis on a TLapha20 (supplier SI Analytics, Weilheim, Germany) titrator. Size exclusion chromatography (SEC) measurements were performed on a Shimadzu system SCL-10A VP (system controller), DGU-14A (degasser), LC-10AD VP (pump), SIL-10AD VP (auto sampler), Techlab (oven), SPD-10AD VP (UV detector), RID-10A (RI detector) and two PSS SDV guard/linear S (5 µm particle size) columns in series (eluent: chloroform/isopropanol/trimethylamine [94/2/4]; flow rate of 1 mL/min at 40 °C) using linear poly(methyl methacrylate) standards. The differential scanning calorimetry (DSC) measurements were performed on a DSC 204 F1 Phoenix from Netzsch under a nitrogen atmosphere with a heating rate of 20 K min^−1^. The thermal gravimetric analysis (TGA) was carried under nitrogen using a Netzsch TG 209 F1 (Selb, Germany). All isothermal titration calorimetry (ITC) experiments were performed using a standard volume Nano ITC (TA Instruments, Hüllhorst, Germany) at 303 K. Solutions was prepared prior utilization by dissolving 2,2’:6’,2”-terpyridine (cell) and the metal salts (syringe) in a mixture of the dry solvents methanol and chloroform (2:1). Blank titrations in the dry solvent were performed and subtracted from the corresponding titration to remove the effect of dilution. The fitting of the measured data was performed NanoAnalyze program from TA instruments (model: independent, TA Instruments, Hüllhorst, Germany). The self-healing scratch tests were performed on an Anton Paar Micro scratch tester MST^3^ (Graz, Austria) on a STeP 4 platform. The instrument was equipped with 10 µm and 50 µm Rockwell C indenters and the optical images were taken with the lenses MPlan N 5×/0.10/FN22 and MPlan N 20×/0.40/FN22. The same set-up was utilized for the Vickers hardness using a Vickers diamond indenter V-K 93 (Anton Paar, Graz, Austria).

The grinding of the samples was performed on a Presi Minitech 250 SP1 (Hagen, Germany) with REFLEX NAC TYPE M 250mm P2500, P1200, and P600, as well as REFLEX PAD-MAG 250 mm TFR and RAM with diamond suspension gel polycrystalline 1 µ and 3 µ, respectively. The Polymer samples were embedded in epoxy resin consisting of Epoxy Resin L and Hardener CL from R&G Faserverbundstoffe GmbH (Waldenbuch, Germany).

### 2.2. Synthesis of the Monomer and Copolymer

#### 2.2.1. Synthesis of 6-(2,2′:6′,2′’-terpyridin-4′-yloxy)-hexan-1-ol (**1**)

According to literature [16], 3.92 g KOH (69.86 mmol) were suspended in 100 mL dry DMSO. The obtained mixture was heated to 40 °C and 17.66 g 1,6-hexanediol (149.43 mmol) was added. Then 4.00 g 4’-chloro-(2,2’:6’,2′’-terpyridine) (14.97 mmol) was added after 30 min at 40 °C. Subsequently, the mixture was stirred for 4 h at 40 °C. Afterwards, the solution was poured into 650 mL deionized water and filtrated. The resulting crude white product was washed with deionized water and dried in vacuo (4.99 g, 95%).

Melting point: 95 °C; ^1^H NMR (300 MHz, CDCl_3_): *δ* = 8.69 (d, *J* = 7.5 Hz, 2H), 8.60 (d, *J* = 8.0 Hz, 2H), 8.01 (s, 2H), 7.84 (td, *J* = 7.7, 1.8 Hz, 2H), 7.33 (dd, *J* = 7.5, 1.1 Hz, 2H), 4.24 (t, *J* = 6.1 Hz, 2H), 3.67 (t, *J* = 6.4 Hz), 1.45 – 1.88 (m, 9H) ppm; ^13^C NMR (75 MHz, CDCl_3_): *δ* = 167.3, 157.1, 156.2, 149.0, 136.8, 123.8, 121.3 107.4, 68.1, 62.9, 32.7, 28.9, 25.8, 25.4 ppm; EA (C_21_H_23_N_3_O_3_): Anal. calculated: C: 71.62, H: 6.31, N. 12.53; found: C: 71.58, H: 6.26, N: 11.85.

#### 2.2.2. Synthesis of 6-(2,2’:6’,2′’-terpyridin-4′-yloxy)-hexyl methacrylate (**2**)

According to literature [16], **1** (1.5 g, 4.3 mmol) was dissolved dichloromethane (50 mL) under nitrogen atmosphere at 0 °C. Afterwards, triethylamine (2 mL, 14.2 mmol) was added and the solution was cooled for a further 30 min. Subsequently, 0.63 mL methacryloyl chloride (6.5 mmol) was slowly added. The mixture was cooled for 2 h at 0 °C. Afterwards, the solution was stirred for 21 h at room temperature. The solvents were removed *in vacuo* and the residue was dissolved in chloroform. The organic phase was washed with deionized water (3 × 100 mL) and was dried using Na_2_SO_4_. The raw product was purified by silica gel chromatography (CHCl_3_) and a white solid was obtained (1.39 g, 77%).

Melting point: 95 °C; ^1^H NMR (300 MHz, CDCl_3_): *δ* = 8.72 – 8.68 (m, 2H), 8.65 (d, *J* = 8.0, 2H), 8.05 (s, 2H), 7.89 (td, *J* = 7.8, 1.8, 2H), 7.37 (ddd, *J* = 7.5, 4.8, 1.1, 2H), 6.10 (s, 1H), 5.57 (s, 1H), 4.26 (t, *J* = 6.5, 1H), 4.18 (t, *J* = 6.6, 1H), 1.97 – 1.85 (m, 5H), 1.81 – 1.67 (m, 2H), 1.64 – 1.48 (m, 4H) ppm; ^13^C NMR (75 MHz, CDCl_3_): *δ* = 167.6, 167.4, 156.9, 156.0, 148.9, 137.0, 136.5, 125.3, 123.9, 121.5, 107.5, 68.1, 64.7, 28.9, 28.6, 25.8, 25.7, 18.4 ppm; EA (C_25_H_273_N_3_O_3_): Anal. calculated: C: 71.92, H: 6.52, N. 10.06; found: C: 71.91, H: 6.64, N: 9.99.

#### 2.2.3. Synthesis of Poly-BMA-*stat*-tpyMA (**P1**)

According to typical RAFT procedure [15] in a 20 mL microwave vial, a 2 M solution was prepared by dissolving **2** (1.174 g, 2.813 mmol, 10 mol%) and 4.00 g of butyl methacrylate (28.13 mmol) in 13.371 mL dry *N,N*-dimethylformamide (DMF). Afterwards, the exact volumes of the stock solution of the RAFT agent 2-cyano-2-propyl benzodithioate (CPDB; stock solution: 46.66 mg in 2 mL DMF; 1.351 mL were added) and the initiator azo-*bis*(isobutyronitrile) (AIBN; stock solution: 8.469 mg in 1 mL DMF; 0.749 mL were used) were added. The [M]:[CPDB] ratio was 150:1 and the ratio of [CPDB]:[AIBN] was 4:1. After purging with nitrogen for 60 min, the reaction was performed at 70 °C for 17 h. After cooling to room temperature, the solution was precipitated into 500 mL of cold methanol twice. After drying *in vacuo*, a pink solid was obtained (3.942 g).

^1^H NMR (300 MHz, CDCl_3_): *δ* = 8.68 (d, *J* = 4.3 Hz, 2H), 8.61 (d, *J* = 7.8 Hz, 2H), 8.00 (s, 2H), 7.84 (t, *J* = 7.2, 1.8 Hz, 2H), 7.37–7.28 (m, 2H), 4.22 (t, *J* = 6.1 Hz, 2H), 3.93 (bs, 24 Hz), 2.09–0.70 (m, 152H) ppm; SEC (chloroform: isopropanol: trimethylamine; standard: poly(methyl methacrylate)): M_n_ = 20,000 g mol^−1^, M_w_ = 24,000 g mol^−1^, Ð = 1.13; TGA: *T_d_* = 290 °C; DSC: *T_g_* = 22 °C.

### 2.3. Synthesis of the Metallopolymers

#### 2.3.1. General Procedure of the Synthesis of the Metallopolymers

The polymer **P1** (0.5 g) was dissolved in 5 mL chloroform. The metal salts (one molar third for each terpyridine moiety) were dissolved in 2 mL methanol and added to the polymeric solution. After stirring the combined mixture at room temperature for 1 h the solvents were evaporated and the residual metallopolymer was dried under reduced pressure.

#### 2.3.2. Synthesis of Poly-BMA-*stat*-tpyMA[Eu]_0.33_ (**P2**)

A solution of 41 mg Eu(NO_3_)_3_·5H_2_O (0.096 mmol) in 2 mL methanol was added to the polymeric solution, consisting of **P1** (0.5 g) and 5 mL chloroform. After stirring and drying pink solid was obtained (470 mg).

^1^H NMR (300 MHz, CDCl_3_): *δ* = 8.67 (s, 2H), 8.60 (d, *J* = 7.6 Hz, 2H), 8.00 (s, 2H), 7.84 (t, *J* = 7.0 Hz, 2H), 7.35 – 7.28 (m, 2H), 4.17 (s, 4H), 3.93 (bs, 46H), 2.05 – 0.77 (m, 168H); SEC (chloroform: isopropanol: trimethylamine; standard: poly(methyl methacrylate)): M_n_ = 22,000 g mol^−1^, M_w_ = 24,600 g mol^−1^, Ð = 1.12; TGA: *T_d_* = 280 °C; DSC: *T_g_* = 43 °C.

#### 2.3.3. Synthesis of Poly-BMA-*stat*-tpyMA[Tb]_0.33_ (**P3**)

A solution of 42 mg Tb(NO_3_)_3_·5H_2_O (0.096 mmol) in 2 mL methanol was added to the polymeric solution, consisting of **P1** (0.5 g) and 5 mL chloroform. After stirring and drying pink solid was obtained (525 mg).

^1^H NMR (300 MHz, CDCl_3_): *δ* = 8.66 (m, 4H), 8.02 (s, 2H), 7.84 (s, 2H), 7.31 (s, 2H), 4.23 (s, 4H), 3.90 (bs, 36H), 1.99 – 0.73 (m, 185H); SEC (chloroform: isopropanol: trimethylamine; standard: poly(methyl methacrylate)): M_n_ = 22,900 g mol^−1^, M_w_ = 24,000 g mol^−1^, Ð = 1.05; TGA: *T_d_* = 284 °C; DSC: *T_g_* = 64 °C.

#### 2.3.4. Synthesis of Poly-BMA-*stat*-tpyMA[Dy]_0.33_ (**P4**)

A solution of 41 mg Dy(NO_3_)_3_·nH_2_O (0.096 mmol) in 2 mL methanol was added to the polymeric solution, consisting of **P1** (0.5 g) and 5 mL chloroform. After stirring and drying pink solid was obtained (470 mg).

^1^H NMR (300 MHz, CDCl_3_): *δ* = 8.71 (s, 4H), 8.02 (s, 2H), 7.86 (s, 3H), 7.30 (d, 2H), 4.23 (bs, 4H), 3.90 (bs, 68H), 1.64 (m, 190 H); SEC (chloroform: isopropanol: trimethylamine; standard: poly(methyl methacrylate)): M_n_ = 22,400 g mol^−1^, M_w_ = 24,200 g mol^−1^, Ð = 1.08; TGA: *T_d_* = 277 °C; DSC: *T_g_* = 45 °C.

### 2.4. Preparation of Samples for Microscopy and Mechanical Investigation

In order to achieve homogeneous samples, 250 mg of each metallopolymer was heated to 100 °C for 1 h. Afterwards, each polymer was directly hot pressed in small cylinders with diameters of 8 mm and heights of about 2 to 3 mm. These were embedded on a surface of an epoxy resin that was utilized as the sample holder. A planar surface was achieved through grinding and polishing the sample cylinder.

## 3. Results and Discussion

This study focuses on the self-healing ability of reversible metal–ligand interactions in polymers. The metallopolymers based on terpyridine complexes with the lanthanoides europium (III), terbium (III), and dysprosium (III) in the side chain of a poly(butyl methacrylate) backbone.

**Scheme 1:**
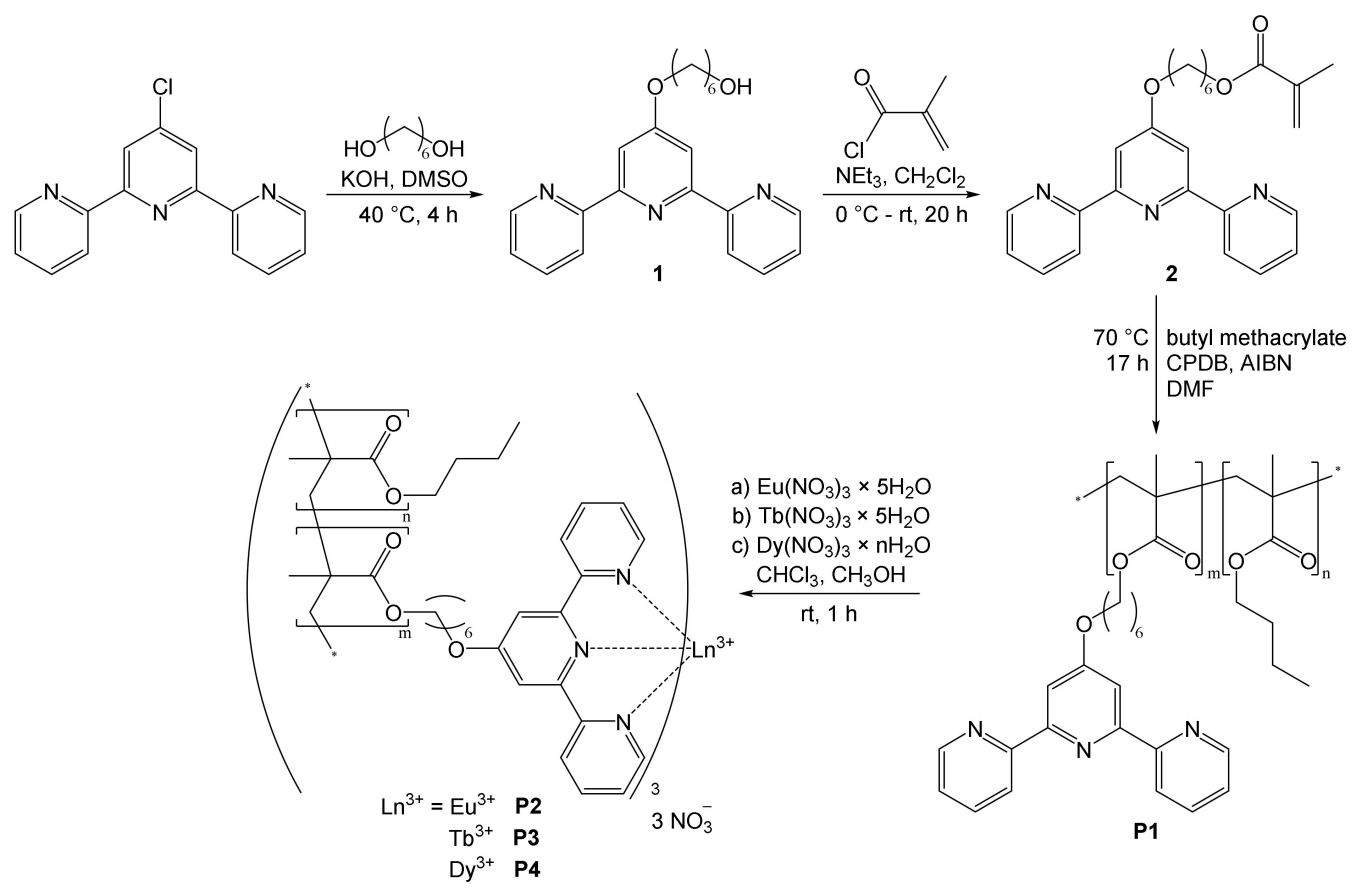
Schematic representation of the synthesis of the metallopolymers **P2** to **P4**.

The synthesis of the metallopolymers is presented in Scheme 1 and was performed according to Bode et al. [22] The first step was an ether synthesis of 4′-chloro-2,2′:6′,2′′-terpyridine and 1,6-hexanediol to **1** (yield 79%) followed by esterification resulting in a terpyridine methacrylate **2** (yield 87%). The monomer **2** was copolymerized with butyl methacrylate (BMA) applying the reversible addition fragmentation chain-transfer (RAFT) polymerization in a ratio of 1:10. The controlled polymerization technique yielded a molar mass M_n_ of 20,000 g mol^−1^ and a dispersity (Ð) of 1.10 (see Table 1). The ratio of the monomers BMA to **2** in copolymer **P1** was found to be 12:1. Afterwards, **P1** was converted with lanthanoidic metal ions europium (III), terbium (III), or dysprosium (III), respectively, by stirring both compounds in a mixture of chloroform and methanol. As a counter ion, nitrate was chosen in all cases in order to exclude the effect of the counter ion and to study the effect of the metal center. Nitrate was previous found to not complex metal ions in metallopolymers and can, therefore, be neglected as an impact on the healing [15]. After the addition of the metal salts, the metallopolymers were also investigated by size exclusion chromatography (SEC) and showed an increased M_n_. The Eu (III)-containing **P2** features a M_n_ of 22,000 g mol^−1^, **P3** (Tb (III) based) a M_n_ of 22,900 g mol^−1^ and the Dy (III)-containing polymer (**P4**) has a molar mass (M_n_) of 22,400 g mol^−1^ (see Table 1). The dispersity indices are relatively low. The condition of the size exclusion chromatography presumably results in a partial decomplexation and a breaking of the network structure. Therefore, it is assumed that only some metals are incorporated into the polymeric structure leading to such low values of the dispersity. Similar phenomena are known in literature [23].

Besides NMR spectroscopy, the polymers were characterized by measuring the glass transition temperatures (*T_g_*) (for DSC-curves see Supporting Information – Figures S1–S3)). Thus, the crosslinking of the polymers by the metal ions increased the glass transition temperature (*T_g_*) of **P1** that is found to be 22 °C. The *T_g_* of **P2** is increased to 43 °C, while the *T_g_* of **P4** is slightly higher with 45 °C (see Table 1). On the other hand, **P3** shows a much higher signal at 61 °C in the third heating cycle as well as small peak at 68 °C in the second heating cycle. The absence in the later heating cycle could be a hint that some impurity in the sample is combusted. Nevertheless, **P3** revealed the highest glass transition temperature among the investigated polymers, which can be due to more stable metal ligand interactions within this metallopolymer compared to complexes of europium (III) or dysprosium (III). Furthermore, all metallopolymers featured an increased *T_g_*-value compared to the ligand-containing copolymer **P1** indicating a successful crosslinking by metal–ligand interactions. Additionally, the polymers were investigated through TGA whereupon it was found that the polymer **P1** is stable up to 290 °C and the *T_d_* of the metallopolymers decreases to around 280 °C (see Table 1 and Supporting Information for TGA-curves – Figures S4–S6).

**Figure 1:**
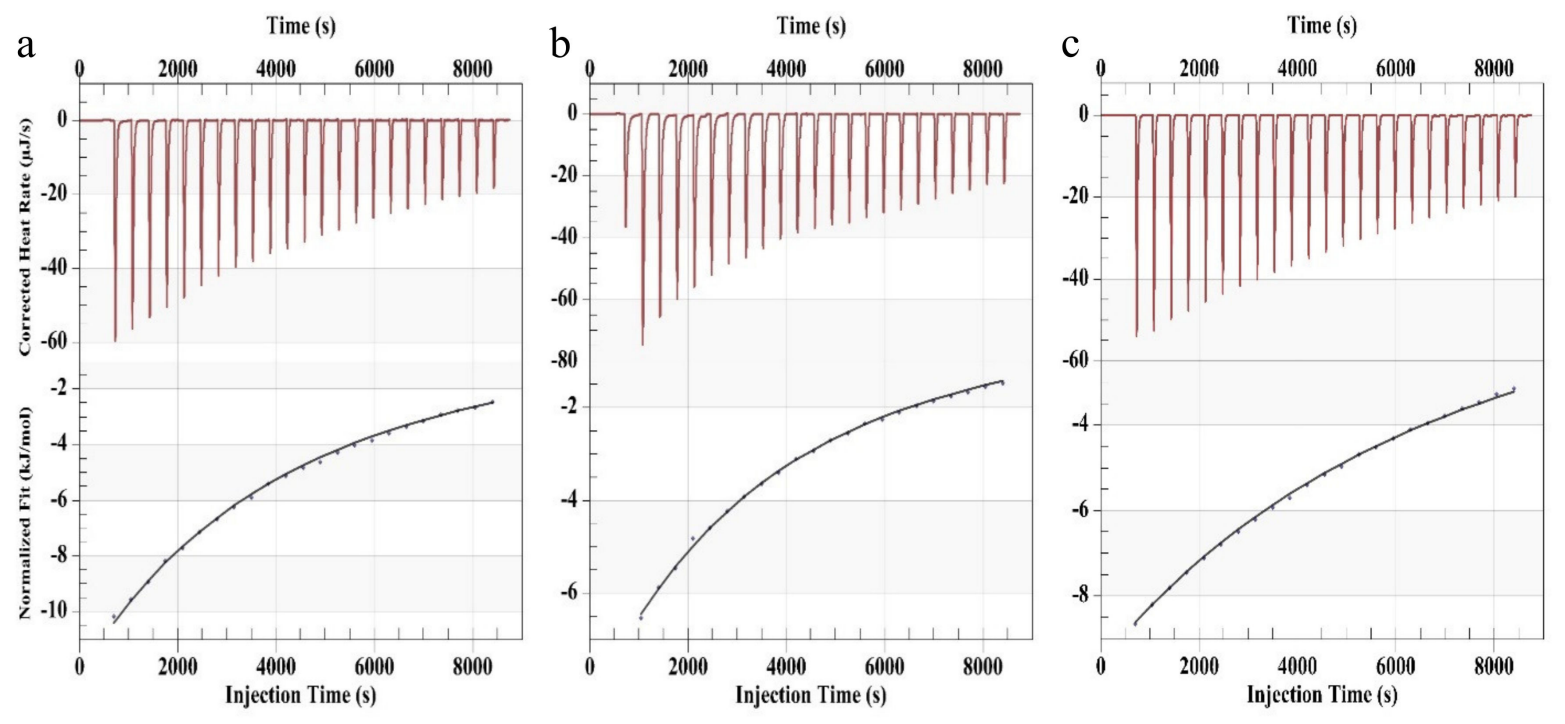
Isothermal titration calorimetry (ITC) curves of the titration of (**a**) Eu(NO_3_)_3_·5H_2_O, (**b**) Tb(NO_3_)_3_·5H_2_O or (**c**) Dy(NO_3_)_3_·nH_2_O, respectively, in the syringe to 2,2’:6’,2”-terpyridine in the cell.

In order to investigate the different complexation strengths of the metal complexes of the respective polymers, isothermal titration calorimetry (ITC) was applied. All utilized lanthanoid ions feature nine coordinating positions and, thus, should enable the complexation of three 2,2’:6’,2”-terpyridine moieties. This complexation ratio could be verified by ITC. The results are presented in Figure 1 and summarized in Table 2. In case of Tb(NO_3_)_3_·5 H_2_O as well as Dy(NO_3_)_3_·nH_2_O a 20 mM solution was prepared, whereas the concentration of Eu(NO_3_)_3_·5H_2_O was increased to 50 mM due to too low intensity during the measurement. The concentration of the ligand 2,2’:6’,2”-terpyridine was one tenth of the concentration of the associated salt solutions. After dissolving, the metal salts were titrated from a syringe into a solution of 2,2’:6’,2”-terpyridine in the measurement cell. The ITC experiment confirmed the ratio of about 0.33 to 1 of the metal ion to the ligand and, thus, the expected complexation configuration. Furthermore, the association (K_a_) and dissociation (K_d_) constants in solution could be determined which are also important factors for self-healing systems. Terbium (III) features the lowest dissociation constant K_d_ (0.0057 M) while those of Eu (III) (0.01023 M) and Dy (III) (0.01249 M) are quite similar. The same trends could be calculated for the association constants K_a_ due to their equation (K_d_ = 1/K_a_). In particular, Dy (III) features a K_a_ of 80.06 M^−1^, followed by Eu (III) (97.77 M^−1^) and Tb (III) revealed the highest K_a_ (175.50 M^−1^). Consequently, the terpyridine complex with Tb (III) is the most stable one, which can be correlated with the high *T_g_*_-_value of **P3**. Thus, the high stability of the metal complexes results in a better network stability of the polymer, a lower mobility and, consequently, in a higher *T_g_*-value. The highest K_d_ of the terpyridine Dy (III) complex makes this metal ligand interaction most labile among these three system. Next to the complexation constants further thermodynamic values such as the changes of enthalpy (ΔH) as well as entropy (ΔS) could be determined which are listed in Table 2.

Those results were measured in solution in a low concentration, which provides useful hints on the complexation behavior, however, the self-healing ability of the metallopolymers has to be studied in detail due to further effects in the solid state.

For this purpose, cylindrical homogeneous samples of the metallopolymers were prepared by hot pressing, embedding into an epoxy resin and polishing of the surface. These samples were utilized to determine the Vickers hardness of the metallopolymers. The metallopolymer **P2** features the highest Vickers hardness with 7.34 (see Table 3), while **P4** shows the lowest Vickers hardness of 6.84. Thus, the weakest complexation in solution also results in the lowest hardness of the polymer network. Nevertheless, this correlation is not generally suitable, since the most stable complexes (Tb (III)) did not result in the hardest metallopolymer.

**Figure 2:**
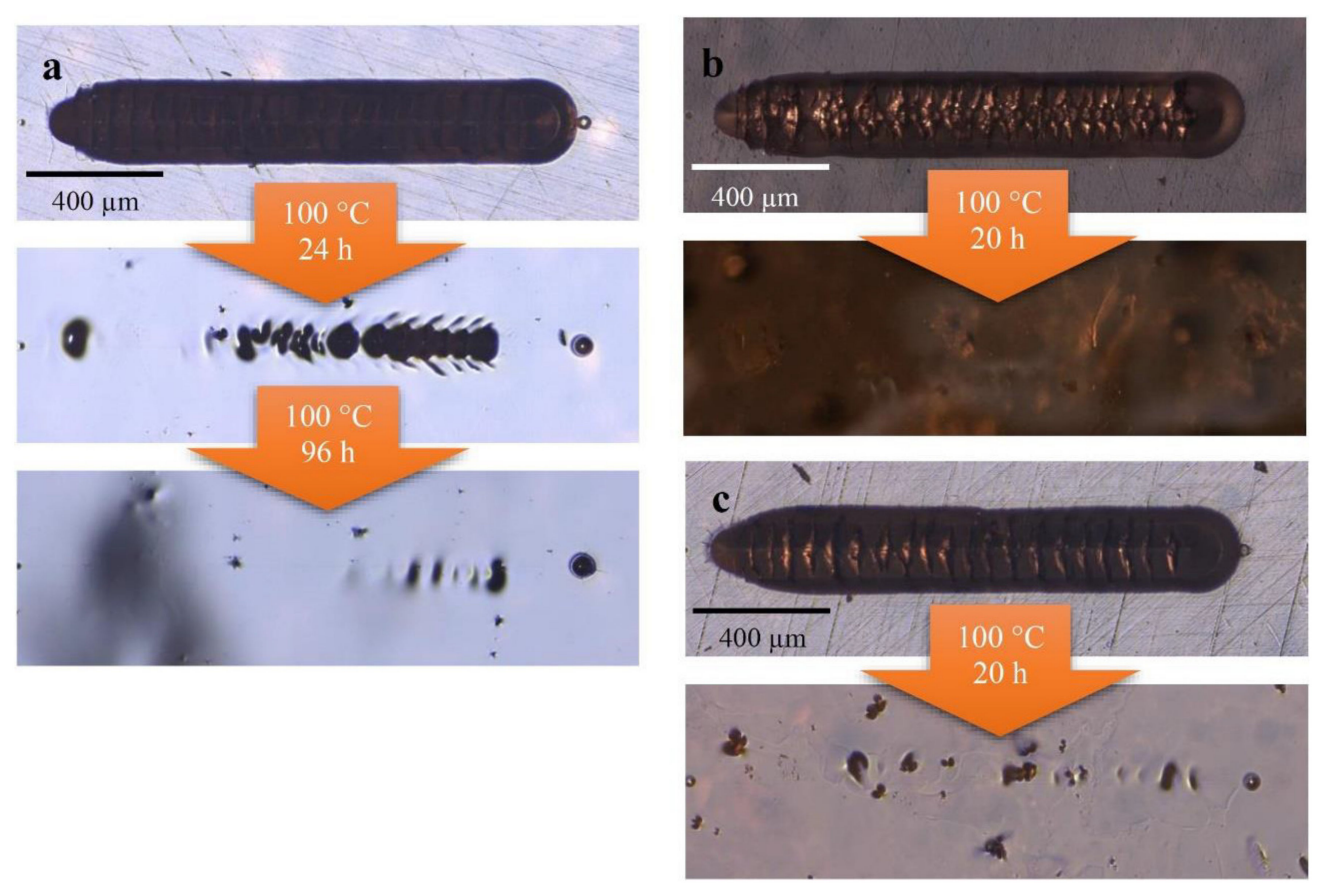
Healing experiments of the metallopolymers **P2** (**a**), **P3** (**b**), and **P4** (**c**); microscopic pictures of the scratch before and after the annealing are presented.

Furthermore, these samples were also utilized to investigate the self-healing ability of the metallopolymers. The self-healing experiments were performed with an indenter that enables defined scratches in length and width by applying a precise force of 3000 mN. The applied length of the scratches was 2000 µm and the depth depended on the hardness of the metallopolymers. Pictures of the scratches are shown in Figure 2. The scratched samples were annealed at 100 °C. After 24 h, the metallopolymer **P2** was not healed completely and, thus, it was annealed for a further 96 h after which an almost complete healing was achieved. **P3** and **P4** featured better healing properties, which can be seen in Figure 2. **P3** is healed completely after 20 h and at **P4** revealed only some small defects.

**Figure 3:**
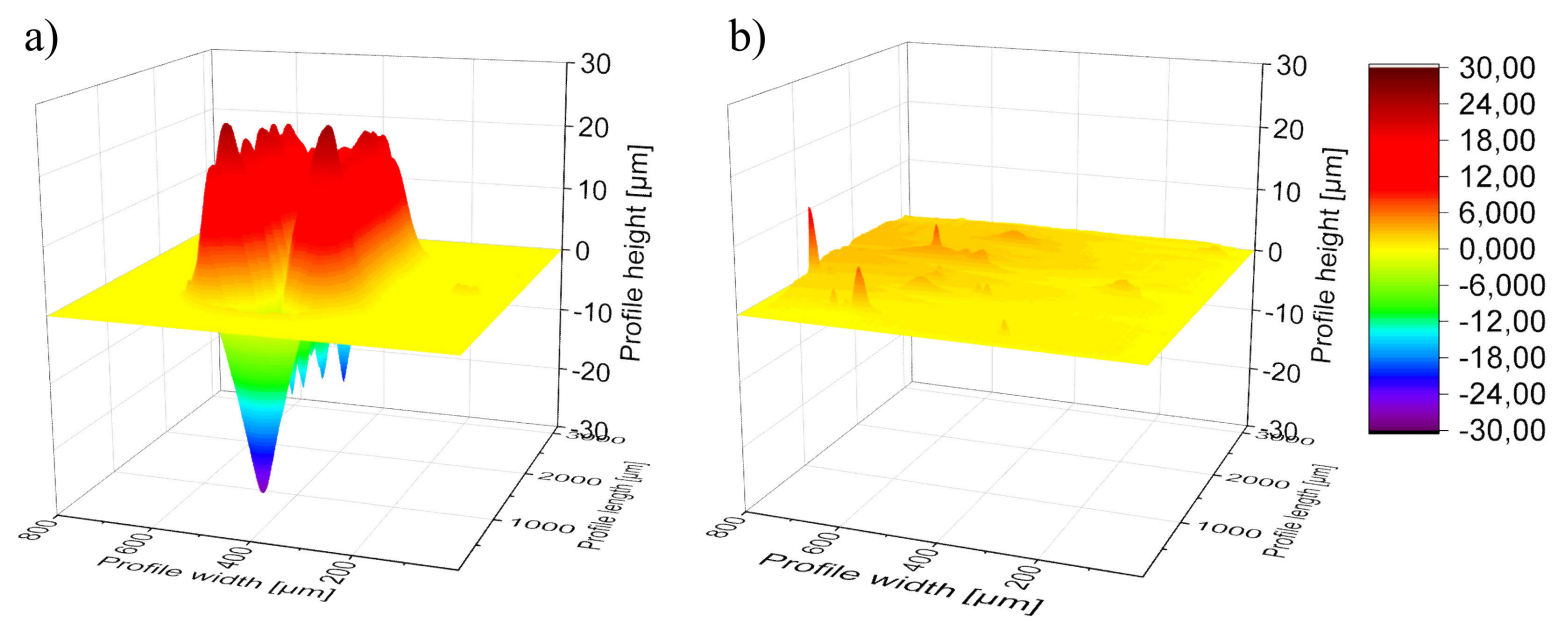
Surface profile of (**a**) the scratch and (**b**) after 20 h healing at 100 °C of metallopolymer **P4**.

Afterwards, height profiles of the scratches, those of the healed metallopolymers as well as the partial healing in case of **P2** after 24 h were measured using a Vickers indenter with an applied force of 3 mN. The profiles were measured every 20 µm. The height profiles were combined in order to convert them into a surface profile. Figure 3 shows these three-dimensional presentations of the scratch as well as of the healed surface of **P4** (**P2** and **P3** are presented in the Supporting Information, Figures S7 and S8). All three scratched surfaces show pushed edges along the scratches and the scratches features deeper cracked segments along their valleys. This could be caused by the relatively high hardness and stiffness of the metallopolymers, which results in the emergence of cracked plates. However, after the healing period of 20 h at 100 °C **P4** shows a more or less flat surface with some defects and little raised segments, which can be attributed to decreased viscosity at higher temperatures resulting in a more inhomogeneous surface compared to the polished surfaces before. In the case of **P2**, the scratch was healed, however, some humps along the previous scratch can be detected, which can be assigned as “scars”. On the other hand, **P3** features an uneven surface after the healing. However, the crack cannot be detected anymore.

Consequently, the surfaces from the combined height profiles supported the analyzation of the scratch healing and identified more or less complete healing of all samples. Furthermore, this method demonstrated the changes on the surface of the samples that become more uneven during the healing process.

Regarding the metallopolymers of this work, **P2** (Eu (III)-based) features the worst healing ability, which may be attributed to the highest Vickers hardness among these three polymers. The best healing was observed for **P3** (Tb (III)-based) and **P4** (Dy (III)-based), which both are not as hard as **P2**. Nevertheless, this finding is contrary to the ITC-measurements in which the terpyridine–terbium (III) complex was found to have the lowest K_d_, which is approximately half of the others. Accordingly, the K_a_ of this complex is the highest among the investigated complexes and, thus, the metal complex should be the most stable one. Consequently, it can be assumed that the complex stability seems to not be the most important factor for the self-healing behavior and other effects are also important. Thus, a better correlation to the hardness of the materials can be drawn. Nevertheless, these influences of the metal–ligand interaction on the solid state properties should be studied further, since a good correlation to the thermal properties (*T_g_*-values) could be revealed.

By comparing the metallopolymers of this work with similar polymers based terpyridine platinum (II) complexes, which feature more stable complexes, a slightly decreased healing efficiency is observed resulting in longer healing times [16]. Furthermore, the metallopolymers **P2**–**P4** exhibit a higher “local” crosslinking density due to the metal to ligand ratio of 1 to 3 and, thus, steric hindrance may play a more important role in the self-healing efficiency compared to other systems with d-metals.

## 4. Conclusions and Outlook

Within the current study, we synthesized metallopolymers based on terpyridine complexes with the lanthanide ions europium (III), terbium (III), and dysprosium (III) in order to show their self-healing ability. For this purpose, a well-known copolymer was utilized featuring butyl methacrylate as well as a terpyridine-containing methacrylate as monomers. The later ones enable a crosslinking of three tridentate ligands through one metal ion. ITC investigations confirmed this ratio as well as revealed thermodynamic and kinetic properties of the metal–ligand interaction. According to these results, the terpyridine complex of terbium (III) is the most stable followed by europium (III) and dysprosium (III). Based on these properties in solution, solid samples of the metallopolymers were prepared that were utilized for measurements of Vickers hardness, in which **P2** showed the highest hardness and the Dy(III)-containing **P4** the lowest. In the end, the self-healing ability was investigated, in which next-to-scratch tests also surface calculations based on height profiles that were performed. In conclusion, **P2** revealed the worst self-healing ability, which can be attributed to the highest hardness of the metallopolymer. However, **P3** and **P4** showed similar self-healing abilities, which can also be correlated to their similar hardness values. Nevertheless, there seems to be no concrete correlation between the complex stability in solution and the healing behavior in the solid state.

These metallopolymers show the potential for further investigations. In particular, the photoluminescence can be utilized to gain a deeper understanding on the molecular scale of the self-healing process through fluorescence spectroscopy. Therefore, the metal complexes can be utilized as sensor systems for healing as described previously for other systems [24].

## Figures and Tables

**Table 1:** Summary of the properties of the polymer **P1** to **P4** measured in size exclusion chromatography (SEC) (chloroform: isopropanol: trimethylamine; standard: poly(methyl methacrylate), differential scanning calorimetry (DSC) and thermal gravimetric analysis (TGA).

Polymer	M_n_ [g mol^−1^]	M_w_ [g mol^−1^]	Ɖ	T_g_ [°C]	T_d_ [°C]
**P1**	20,000	24,000	1.13	22	290
**P2**	22,000	24,600	1.12	43	280
**P3**	22,900	24,000	1.05	64	284
**P4**	22,400	23,900	1.09	45	277

**Table 2:** Summary of the complexation values after isothermal titration calorimetry of Eu(NO_3_)_3_·5H_2_O, Tb(NO_3_)_3_·5H_2_O, as well as Dy(NO_3_)_3_·nH_2_O to 2,2’:6’,2”-terpyridine.

Variable	Eu(NO_3_)_3_·5 H_2_O	Tb(NO_3_)_3_·5 H_2_O	Dy(NO_3_)_3_·n H_2_O
c_syringe_ [mM]	51.17	21.62	20.61
c_cell_ [mM]	5.429	2.046	2.046
K_d_ [M]	0.0102	0.0057	0.0125
K_a_ [1/M]	97.77	175.50	80.06
N	0.330	0.308	0.331
ΔH [kJ/mol]	−59.8	−128.3	−184.3
ΔS [J/mol·K]	−159.0	−380.1	−571.6

**Table 3:** Summary of Vickers hardnesses of metallopolymers **P2** to **P4**.

Metallopolymer	HV	d_1_	d_2_
**P2**	7.34	49.276	52.224
**P3**	6.97	51.496	51.566
**P4**	6.84	52.274	51.470

## Supplementary Materials

The following are available online at https://www.mdpi.com/2073-4360/12/4/838/s1.
