# Supplementary material for: Lanthanoids Goes Healing: Lanthanoidic Metallopolymers and Their Scratch Closure Behavior
Source: Polymers (Basel). 2020 Apr 6;12(4):838. doi: 10.3390/polym12040838 (PMC7240633; doi:10.3390/polym12040838)
Supplement: Supplementary file 1 [file polymers-12-00838-s001.pdf]

Supporting information

Lanthanoids goes healing: Lanthanoidic metallopolymers and their scratch closure behavior

Stefan Götz [1,2], Stefan Zechel [1,2], Martin D. Hager [1,2*], Ulrich S. Schubert [1,2*]

[1] Laboratory of Organic and Macromolecular Chemistry (IOMC), Friedrich Schiller University Jena, Humboldtstr. 10, 07743 Jena, Germany; www.schubert-group.de, ulrich.schubert@uni-jena.de; martin.hager@uni-jena.de

[2] Jena Center of Soft Matter (JCSM), Friedrich Schiller University Jena, Philosophenweg 7, 07743 Jena, Germany

Table of content:

1. DSC curves of the polymers S2
2. TGA curves of the polymers S3
3. Animated surface of self-healing experiments S5

1. DSC curves of the polymers

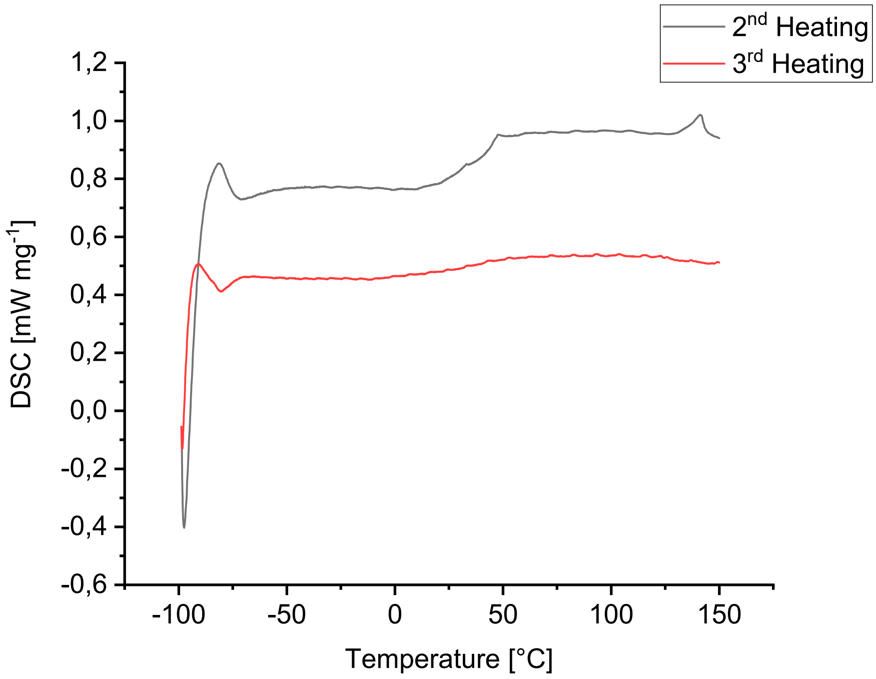

Figure S1: DSC heating cycles of **P2**.

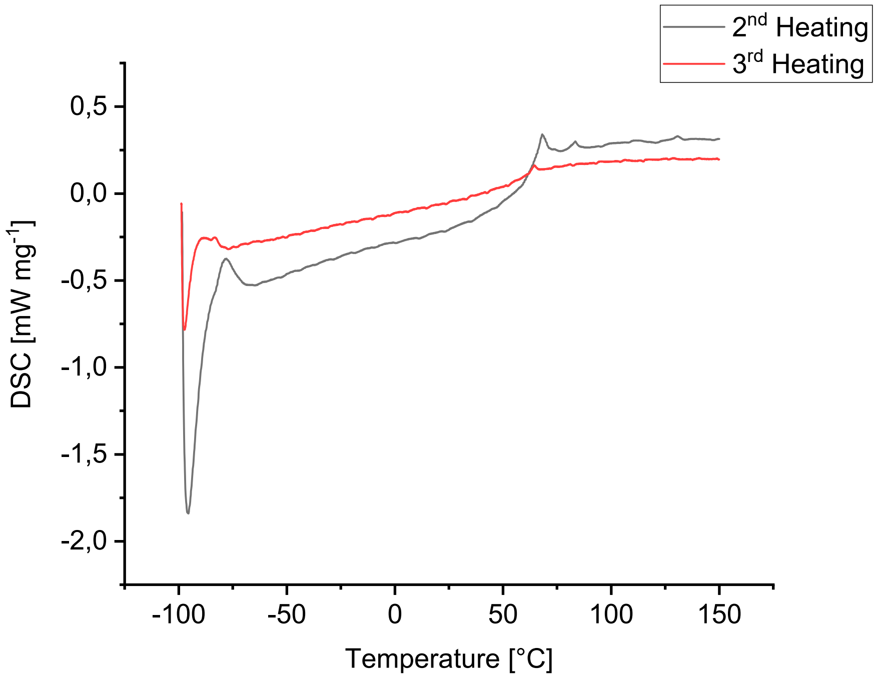

Figure S2: DSC heating cycles of **P3**.

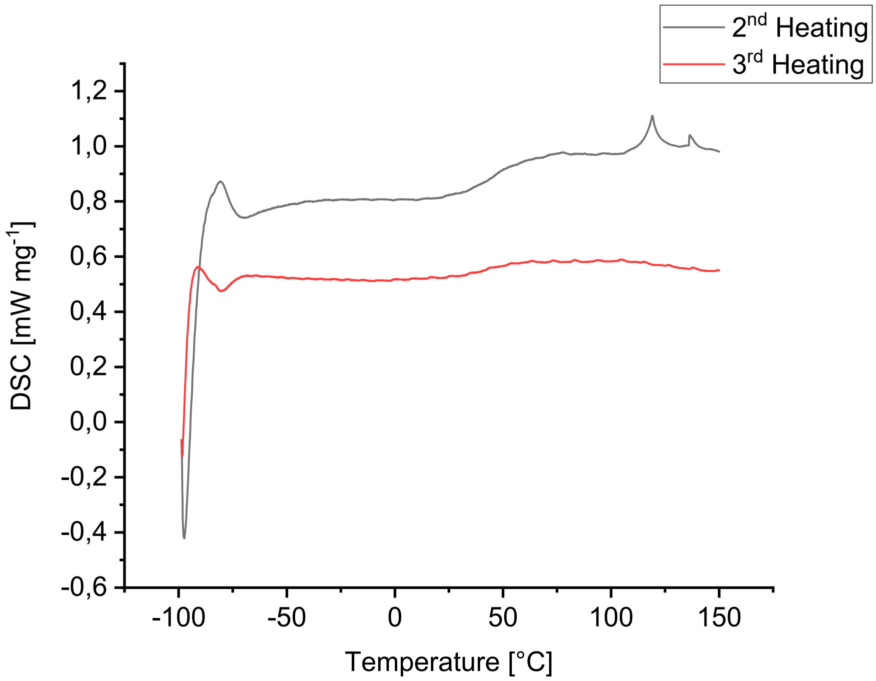

Figure S3: DSC heating cycles of **P4**.

2. *TGA curves of the polymers*

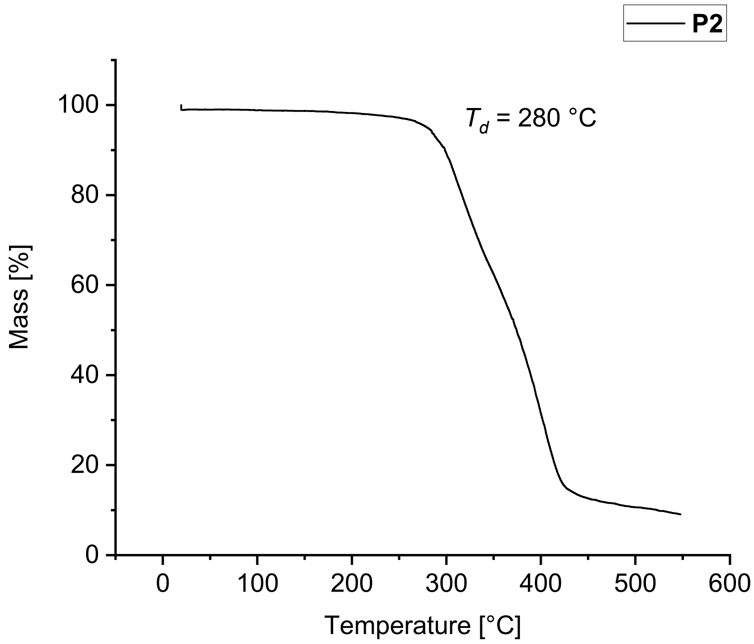

Figure S4: Trace of the TGA measurement of **P2**.

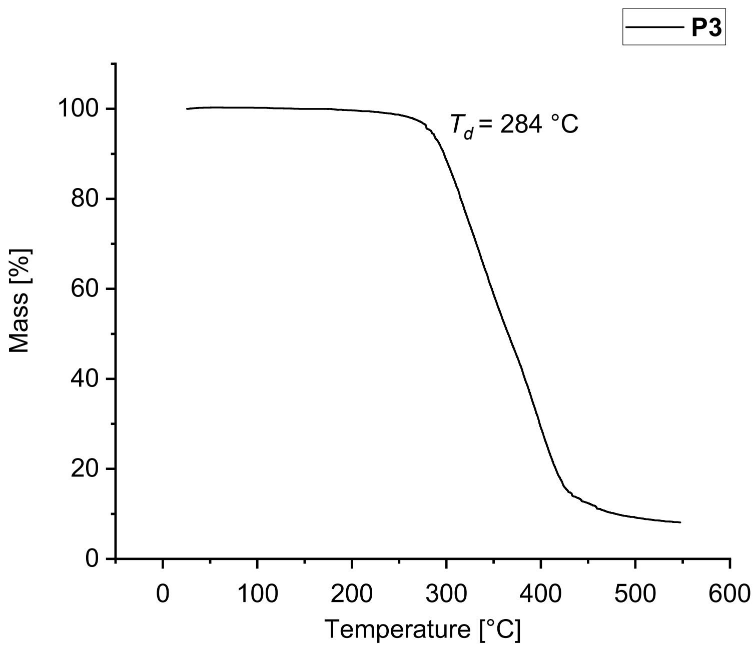

Figure S5: Trace of the TGA measurement of **P3**.

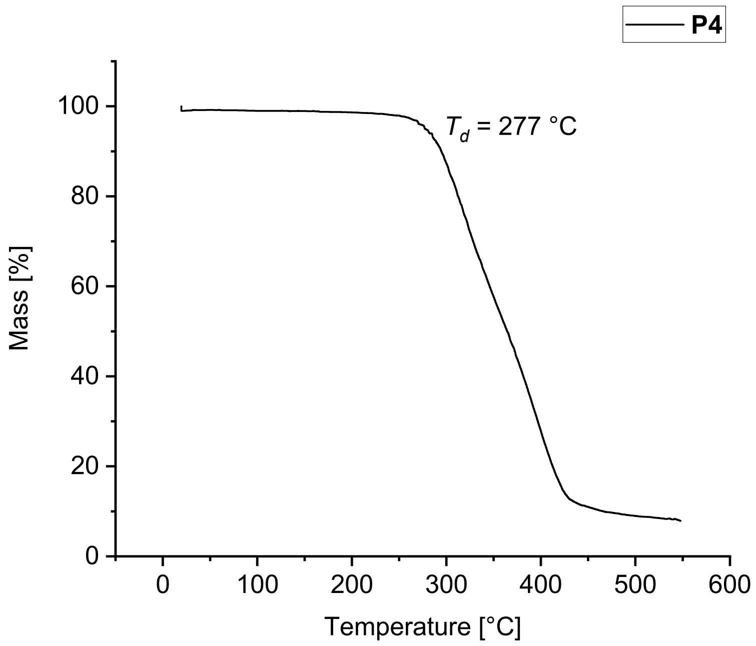

Figure S6: Trace of the TGA measurement of **P4**.

3. *Animated surface of self-healing experiments*

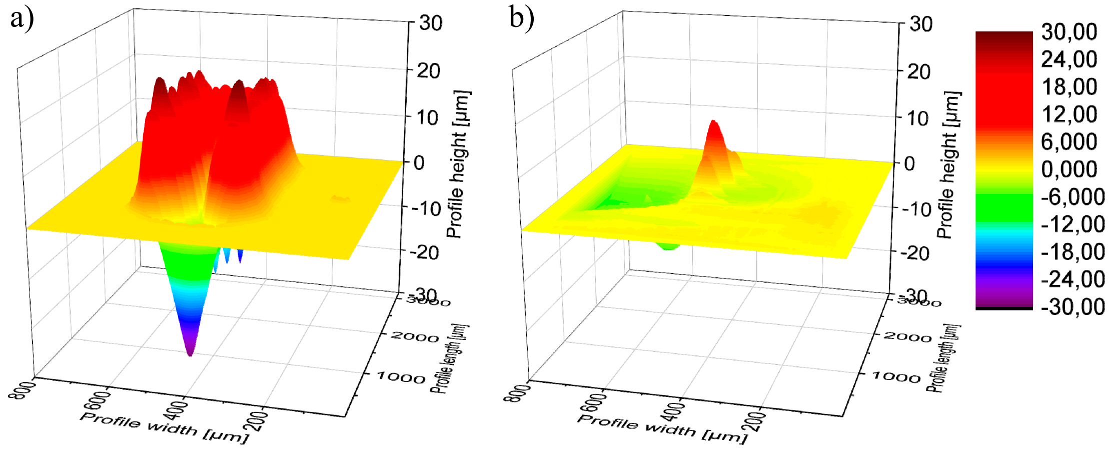

Figure S7: Schematic representation of the surface profile of a) the scratch and b) after 24 h healing at 100 °C (right) of metallopolymer **P2**.

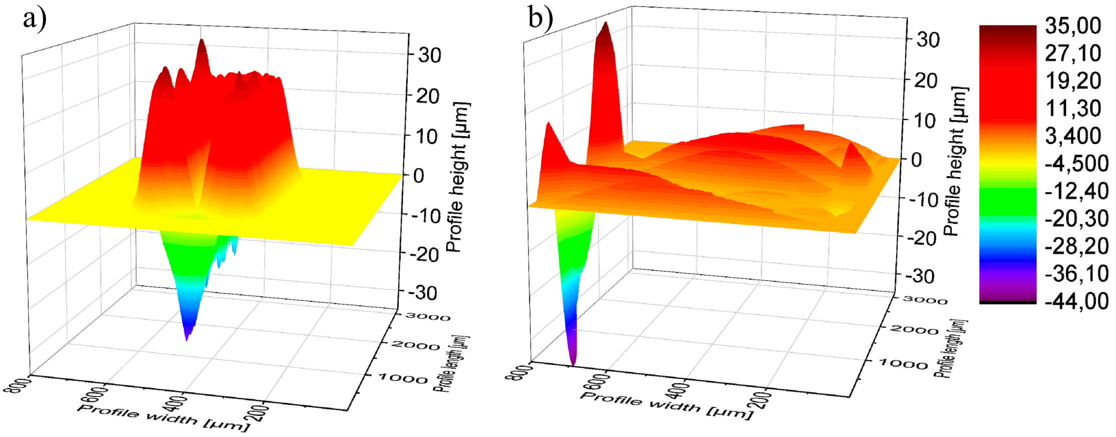

Figure S8: Schematic representation of the surface profile of a) the scratch and b) after 20 h healing at 100 °C of metallopolymer **P3**.
